# Direct measurement of large-scale quantum states via expectation values of non-Hermitian matrices

**DOI:** 10.1038/ncomms10439

**Published:** 2016-01-19

**Authors:** Eliot Bolduc, Genevieve Gariepy, Jonathan Leach

**Affiliations:** 1 Institute of Photonics and Quantum Sciences, School of Engineering & Physical Sciences, Heriot-Watt University, David Brewster Building, Edinburgh EH14 4AS, UK

## Abstract

In quantum mechanics, predictions are made by way of calculating expectation values of observables, which take the form of Hermitian operators. Non-Hermitian operators, however, are not necessarily devoid of physical significance, and they can play a crucial role in the characterization of quantum states. Here we show that the expectation values of a particular set of non-Hermitian matrices, which we call column operators, directly yield the complex coefficients of a quantum state vector. We provide a definition of the state vector in terms of measurable quantities by decomposing these column operators into observables. The technique we propose renders very-large-scale quantum states significantly more accessible in the laboratory, as we demonstrate by experimentally characterizing a 100,000-dimensional entangled state. This represents an improvement of two orders of magnitude with respect to previous phase-and-amplitude characterizations of discrete entangled states.

## Abstract (web summary)

Quantum state tomography is the process of retrieving the values that define a quantum system, but realizing it experimentally can be burdensome. Here, the authors provide an alternative approach via the expectation values of a set of non-Hermitian matrices, and characterize a 100,000-dimensional state.

## Graphical abstract

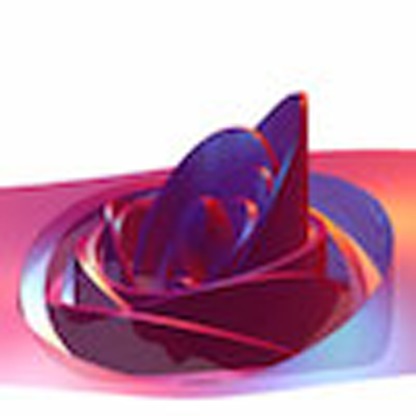

One of the current challenges in the field of computing is harnessing the potential processing power provided by quantum devices that exploit entanglement. Experimental research aimed at overcoming this challenge is driven by the production, control and detection of larger and larger entangled quantum states^1^,^2^,^3^,^4^. However, the task of characterizing these entangled states quickly become intractable as the number of parameters that define a many-body system scales exponentially with the system size. To keep up with the ever-growing quantum state dimensionality, much effort is put into developing efficient characterization methods^5^,^6^,^7^,^8^,^9^,^10^,^11^,^12^,^13^,^14^,^15^,^16^,^17^,^18^,^19^.

Quantum state tomography is the process of retrieving the values that define a quantum system. The process typically involves two steps: (i) gathering an informationally complete set of data and (ii) finding the quantum state most consistent with the data set using post-measurement processing such as the algorithm for maximum-likelihood estimation^20^. Many efficient tomographic methods capitalize on the first step by making simplifying assumptions about the state^11^,^12^,^13^,^14^,^15^,^16^,^17^,^18^,^19^, thus reducing the number of measurements required to uniquely identify it. In particular, tomography via compressed sensing allows one to efficiently reconstruct quantum states based on the fact that low-rank density matrices, that is, quasi-pure states are sparse in a particular basis^15^,^16^,^17^,^21^. Compared with assumption-free tomography, compressive sensing provides a square-root improvement on the required number of measurements^10^. This improvement enabled the reconstruction of the density matrices of a six-qubit state^16^ and a (17 × 17)-dimensional state^17^, the largest phase-and-amplitude measurement of an entangled state reported to date. Although compressed sensing does not make use of maximum-likelihood estimation, it does require non-trivial post-measurement processing.

Recently, Lundeen *et al.*^19^ reported on the direct measurement of a wavefunction using a method that, for the first time, required no complicated post-measurement processing. Their method is based on weak measurements, whereby one weakly couples a quantum system to a pointer state and subsequently performs a few standard strong measurements on the pointer state. The outcome of a weak measurement is known as the ‘weak value', and in the conditions exposed in ref. 19, the weak value is proportional to a given state vector coefficient. The method of Lundeen *et al.* can be used in combination with the assumption that the quantum state at hand is pure, providing the same square-root improvement as compressed sensing. Variations on the original scheme allow measurements of mixed states and increased detection efficiency^22^,^23^,^24^.

An important contribution of the work by Lundeen *et al.* was to link the state vector elements to the expectation value of weak measurements. We take a different approach, and point out that the enabling feature that allows access to the complex state vector is not weak measurement but the use of particular non-Hermitian operators. Although weak measurements provide a way to decompose these non-Hermitian operators, it is not the only suitable approach. Moreover, the introduction of weak values in the measurement procedure adds complexity to the experiment and the formalism that links weak values to measurement outcomes involves an approximation that breaks down in a variety of circumstances^24^,^25^,^26^.

In this paper, we propose an alternative approach to the direct measurement of quantum states that is exact in the case of pure states, proves to be reliable in the presence of noise, and is consistent with results obtained with well-established tomographic techniques. The key principle of our formalism is to decompose the particular non-Hermitian matrices that yield the complex state vector coefficients using only observables. Our method therefore only requires strong measurements, as in standard tomography, while maintaining the directness of weak-value-assisted tomography. The simplicity in both the experimental procedure and post-measurement processing renders our method ideally suited for the characterization of large-scale systems, which can be high-dimensional, many-body or both. We begin by developing the theory on which our method is based and then demonstrate the potential of this scheme by experimentally retrieving the complex coefficients of a (341 × 341)-dimensional entangled state.

## Results

### Theory

Consider a quantum system in a *d*-dimensional Hilbert space, whose state vector


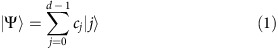


is expanded in the basis {|*j*〉} and where *c*_*j*_ are unknown complex expansion coefficients. To retrieve these coefficients, we introduce the column operators 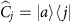, where |*a*〉 is an arbitrary reference vector. Each column operator has an expectation value


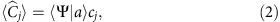


which is proportional to a complex state vector expansion coefficient. Since the value of 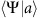 is independent of *j*, we can express the state vector in terms of the column operators up to a phase factor:


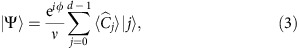


where 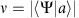 is a normalization constant. We can ignore the phase factor e^*iφ*^ since it bears no physical significance.

Most column operators 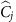 are not Hermitian matrices and are thus not observables. To overcome this apparent constraint, we recognize that any non-Hermitian matrix can be constructed from a complex-weighted sum of Hermitian matrices. Hence, the crucial step to our method is to construct the column operators in terms of measurable quantities: 
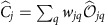
, where *w*_*jq*_ are complex weights and 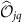 are observables. As a result, this allows us to retrieve any state vector element with a complex-weighted sum of measurement outcomes:


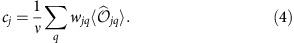


Equation 4 is an exact definition of the pure state vector that is provided in terms of measurable quantities. The above formalism readily applies to a general class of quantum states, including high-dimensional and many-body systems.

As an example, consider the case of a qubit 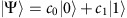 with |*a*〉=|0〉 as the reference vector. The first column operator 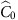 is Hermitian and given by the projector |0〉〈0|. The second column operator 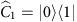 is not Hermitian but can be constructed a number of ways. The first construction—which, as pointed out earlier, is a key part of the weak value formalism—is the complex-weighted sum of Pauli matrices: 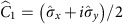, a decomposition that requires two observables, each of which is made of two projectors or eigenvectors. A second decomposition requiring only three projectors is given by


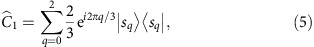


where 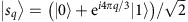 are the states onto which the observables 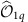 project. In both cases, the qubit state vector is exactly given by 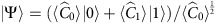.

### Experiment

To demonstrate the power and scalability of our scheme, we apply it to the measurement of a state entangled in greater than 100,000 dimensions. We provide a complete characterization of the spatially entangled two-photon field produced through spontaneous parametric downconversion (SPDC). In general, SPDC can give rise to spatial and frequency correlations between two photons^4^,^27^,^28^,^29^,^30^,^31^,^32^,^33^,^34^,^35^,^36^. The purity of the spatial part of the full state can only be guaranteed if the two types of correlations are completely decoupled, which can be achieved in the collinear regime^27^—see Supplementary Note 1 for a theoretical estimation of our system purity. The consequences of applying our scheme to a quantum state with non-unit purity, which is always the case in the presence of noise, will be discussed below.

We express the spatial part of the entangled state in a discrete cylindrical basis of transverse spatial modes. The azimuthal part of the modes is given by 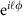, where 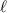 is an integer between −∞ and ∞ and *φ* is the azimuthal angle. This type of phase profile is known to carry 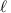 units of orbital angular momentum (OAM). We decompose the radial part of the field with the recently introduced Walsh modes, labelled by the integer *k* ranging from 0 to ∞ (ref. 34). The Walsh modes all have the same Gaussian amplitude envelope, but different *π*-steps radial phase profiles. Combining the OAM modes with the Walsh modes yields a complete basis for coherent two-dimensional images. To perform the characterization of the two-photon spatial field, we consider 31 OAM modes and 11 Walsh modes for each photon. The state vector thus takes the form


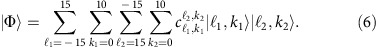


**Figure 1:**
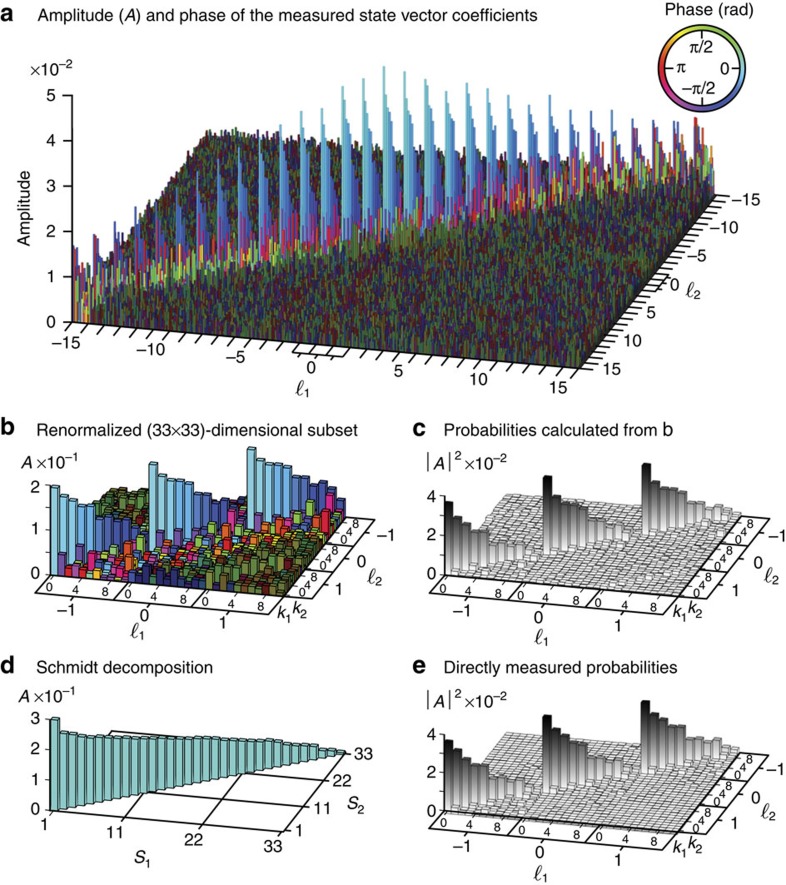
Measured and calculated properties of the two-photon state. (**a**) We illustrate the complex state vector coefficients in matrix format. This representation is similar to that of an optical transfer matrix, where the lateral axes correspond to input and output modes. Here, each lateral axis corresponds to the spatial state of one photon of the pair. The OAM values 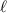 range from −15 to 15. For a fixed OAM value, the radial index *k* ranges from 0 to 10, thus the combined state has 341 × 341 dimensions. The amplitude and phase values of a coefficient are given by the height and colour of a bar, respectively. For clarity, we darken the off-diagonal part. The small subset (**b**) of the state shows the phase gradient across the diagonal elements, which is typical of a Gouy phase shift, a common property of light passing through focus^43^. The  corresponding calculated probability matrix (**c**) is consistent with the directly measured probabilities (**e**). Finally, we calculate the Schmidt decomposition (**d**) of **b**, which gives the joint basis in which the subset can be expressed with the lowest number of modes. The indices *S*_1_ and *S*_2_ correspond to the states of each photon in this basis.

Using the column-operator decomposition described in the Methods section, we sequentially measure all 116,281 coefficients 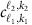, which are shown in Fig. 1a,b. The total Hilbert space dimensionality of this measured state is more than two orders of magnitude larger than any previously reported amplitude-and-phase-characterized discrete entangled state^17^. As a simple verification of the accuracy of our method, we calculate the probabilities associated with each joint mode via the Born rule, 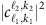, as shown in Fig. 1c. This result is consistent with the directly measured correlation matrix shown in Fig. 1e, showing that we retrieve the correct magnitude of the amplitudes.

To rigorously assess the validity of the directly measured complex quantum state 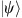, that is, both the amplitudes and the phases, we compare it to the results obtained through full tomography (that is, assumption-free tomography); see Supplementary Note 2 for details of the algorithm used to retrieve the density matrix. As full tomography cannot be performed on a (341 × 341)-dimensional entangled state in a reasonable time, we characterize a (5 × 5)-dimensional subset of the SPDC two-photon state. We perform the comparison in a basis of various OAM modes (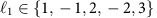, 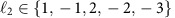) and a fixed radial Walsh mode (*k*_1_=*k*_2_=0). The total number of unknown parameters in the corresponding density matrix is equal to 624. After performing the direct measurement procedure in this basis, we record 8,000 random projective measurements that we break into eight sets of 1,000. For each set, we recover a density matrix *ρ*_exp_ and calculate its purity and the fidelity with the directly measured state 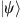; fidelity is defined as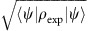. On average, the purity calculation yields (0.96±0.02), and the fidelity gives (0.985±0.004), where the uncertainties correspond to one standard deviation. After reconstruction of a density matrix, we find that the average error between the measured count rates and the count rates predicted by the density matrix is 5.5%. This can be explained by shot noise, the pixelated nature of the spatial light modulator (SLM), and the finite aperture of the optical elements. While we expect unit purity, the 5% noise level accounts for the discrepancy with the measured value.

### Simulation with mixed states

The extremely high fidelity between the tomography results *ρ*_exp_ and the directly measured state 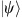 indicates the validity of our approach for quantum state measurements applied to near pure states. To evaluate our method in the context of mixed states, we perform a series of numerical simulations where we vary the rank, purity and dimension of an unknown state *ρ*_sim_, where no sources of noise are added to the simulated measurement outcomes. We apply our direct measurement procedure to these states and calculate the fidelity 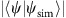, where 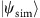 is the eigenvector of *ρ*_sim_ with the largest eigenvalue. For initial states *ρ*_sim_ with purity >0.81, we measure a fidelity >0.99 in at least 99% of the cases. The dependency of this result on the dimensionality of the state is negligible. This result indicates that our direct method enables the extraction of the density matrix primary eigenvector, even for a partially mixed state. Full details of this analysis and the density matrix reconstruction are presented in the Supplementary Note 1.

## Discussion

Knowledge of the amplitude and phase of the state vector elements allows us to perform otherwise inaccessible calculations. As an example, we perform a calculation of the Schmidt decomposition^37^. This is equivalent to the singular value decomposition for the case of optical transfer matrices. The Schmidt decomposition yields a new joint basis, in which the photons are perfectly correlated and where the joint modes have equal phases, as shown in Fig. 1d. When the Schmidt decomposition is applied to the entire state, we calculate a number of Schmidt modes equal to 142; this represents the effective number of independent joint modes contained within the state (the maximum for a (341 × 341)-dimensional state being 341). The Schmidt decomposed two-photon field is a good candidate for the violation of very high-dimensional Bell inequalities^29^. Further details on the Schmidt decomposition can be found in the Supplementary Note 3.

There are a number of approaches to reducing the necessary cost and effort for measuring large-scale quantum states. These include, but are not limited to, developing technologies for mode sorting^38^ and arbitrary unitary transformations^39^,^40^, reducing the required number of measurement settings, and circumventing the requirement for reconstruction procedures. It is clear that there is significant interplay between each of these approaches. The theoretical implementation of an approach that combines the principles of our work with generalized measurements, such as positive operator value measures, is considered in the Supplementary Note 4. The ability to use positive operator value measures in the laboratory relies on the aforementioned technologies. Access to these types of technologies would reduce the overall number of measurement settings to uniquely recover a quantum state. However, such a system requires arbitrary unitary transformations for spatial states, which is in itself an active area of research^38^,^39^,^40^. Given the limitations of mode sorters for very large dimensions, and the practical nature of projective measurements, our scheme provides a simple and elegant method for the characterization of large-scale quantum states.

Our scheme allows direct access to the complex coefficients that define large-scale quantum states. The main result of our work is a novel method for retrieving a state vector coefficient with a complex-weighted sum of strong measurement outcomes. One challenge in reconstructing a quantum state from measurement outcomes lies in data processing; our scheme trades the difficulty of data processing for theoretical analysis before the experiment, that is, finding the measurements one has to perform. We anticipate that our work will have an impact on a number of disciplines, for example, quantum parameter estimation, measurement in quantum computing, quantum information and metrology.

## Methods

### Experiment

**Figure 2:**
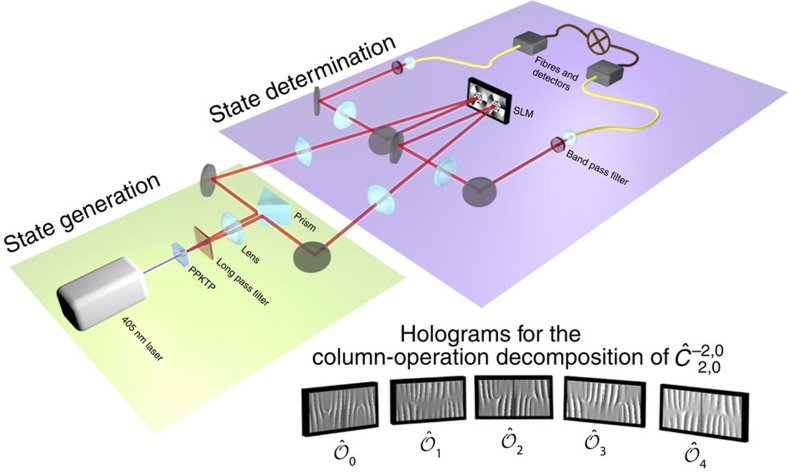
Generation and characterization of a two-photon field. The entangled state is produced via SPDC in a PPKTP crystal and spatially separated by a prism. For the state determination stage, the crystal plane is imaged onto a SLM, which is in turn imaged to the input facet of two single-mode fibres. To make a given projective measurement, we display the corresponding joint mode on the SLM and measure the coincidence rate between the two single-photon avalanche diode detectors. The inset shows the five joint holograms that correspond to the column-operator decomposition of 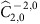. The state vector coefficient 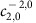 is given by 
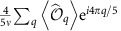
, where the expectation value of a given observable is proportional to the measured count rate when displaying the corresponding hologram.

The two-photon field is generated via SPDC with a 405-nm laser diode pumping a 1-mm-long periodically poled KTP (PPKTP) crystal with 50 mW of power. The experimental set-up is shown in Fig. 2. We separate the two photons with a right-angle prism and image the plane of the crystal to a Holoeye SLM with a magnification of −10. We simultaneously display two holograms, one on each side of the SLM, to control the amplitude and phase profiles of the two photons independently. To make projective measurements of superposition modes, we make use of intensity masking^41^. We image the plane of the SLM with a magnification of −1/2,500 to two single-mode fibres. The combination of the SLM and singles mode fibres allows us to make arbitrary projective measurements. All measurements are performed in coincidence with two single-photon avalanche detectors, with a timing window of 25 ns, an integration time of 1 s for modes outside the diagonal and 20 s for the diagonal elements (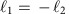 and *k*_1_=*k*_2_). We start an automatic alignment procedure with the SLM every 4 hours to compensate for drift. Including the time it takes to calculate and display a hologram (about 1 s), the entire experiment takes 2 weeks; assumption-free tomography would take more than four centuries at the same acquisition rate. We perform no background subtraction and use the fundamental mode 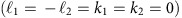 as the reference vector |*a*〉. The count rate of the fundamental mode is approximately 900 coincidences per second and varies by 10% over 24-h periods. To correct for long-term drift, we normalize each outcome to the count rate of the fundamental mode, which we measure before the measurement of each column operator. In standard tomography, the calculation of error bounds on the measured state is not a straightforward task^42^. Here, we can calculate the error bound on a given coefficient with a weighted sum of the detector counts used to retrieve it. For a given state vector coefficient, the errors on the amplitude |*c*_*j*_| and phase arg(*c*_*j*_) are both inversely proportional to the overlap *ν* of the reference vector with the quantum state. To minimize the errors, it is important to choose a reference vector that has a high probability of occurrence within the state—the fundamental mode is the most probable one in our case.

### Two-body column-operator decomposition

To decompose a given state vector coefficient 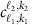 into a set of measurement outcomes, we need to find a projector decomposition of the corresponding column operator 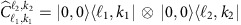, as in equation 4. We numerically find this column-operator decomposition, that is, the complex weights *w*_*q*_ and the observables 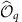, using the differential evolution algorithm (see Supplementary Note 5). By inspection, we find that the corresponding analytical form of the state vector coefficients is given by


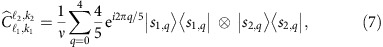


where 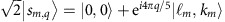 with *m*={1, 2}, and 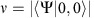 is a normalization constant. This decomposition is only valid when the state of any photon is different from the reference vector, that is, 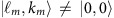. Each coefficient measured with the above column-operator decomposition requires five projective measurements, thus explaining the 5*D*^2^ scaling, where *D* is the Hilbert space dimensionality of a single particle. The protocol scales much more favourably than assumption-free tomography, which requires *D*^4^ projections.

Here, we briefly explain our protocol for measuring the entire SPDC state vector. We measure more than 99% of the coefficients using the decomposition of equation 7. The remaining column operators are the special cases 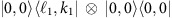 and 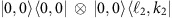, which respectively correspond to a row and a column of the result shown in Fig. 1a. These column operators can be decomposed into only three joint local measurements using the projector |0, 0〉〈0, 0| on one system and a column-operator decomposition similar to that of equation 5 on the other system. Finally, the column operator |0, 0〉〈0, 0|⊗|0, 0〉〈0, 0| is a projector, and its expectation value can be measured in a single experimental configuration.

### Full quantum tomography

We perform full tomography with high count rates in order to achieve high accuracy. We set the magnification between the plane of the SLM and that of the single-mode fibres to 1/400. In this condition, we obtain a count rate of approximately 18,000 counts per second for the fundamental mode and integrate over 1 s for each individual projective measurement. The increase in the count rate of the fundamental mode comes at the price of lower count rates for high order modes. Regarding the full tomography measurements, we take an overcomplete set of 1,000 random projective measurements in a (5 × 5)-dimensional space. To minimize high-frequency components on the SLM, we limit the random superpositions to two-dimensional subsets of the state space.

## Additional information

**How to cite this article:** Bolduc, E. *et al.* Direct measurement of large-scale quantum states via expectation values of non-Hermitian matrices. *Nat. Commun.* 7:10439 doi: 10.1038/ncomms10439 (2016).

## Supplementary Material

Supplementary Information Supplementary Figures 1-6, Supplementary Notes 1- 5 and Supplementary References.
