# Supplementary material for: Direct measurement of large-scale quantum states via expectation values of non-Hermitian matrices
Source: Nat Commun. 2016 Jan 19;7:10439. doi: 10.1038/ncomms10439 (PMC4735685; doi:10.1038/ncomms10439)
Supplement: Supplementary Information — Supplementary Figures 1-6, Supplementary Notes 1- 5 and Supplementary References. [file ncomms10439-s1.pdf]

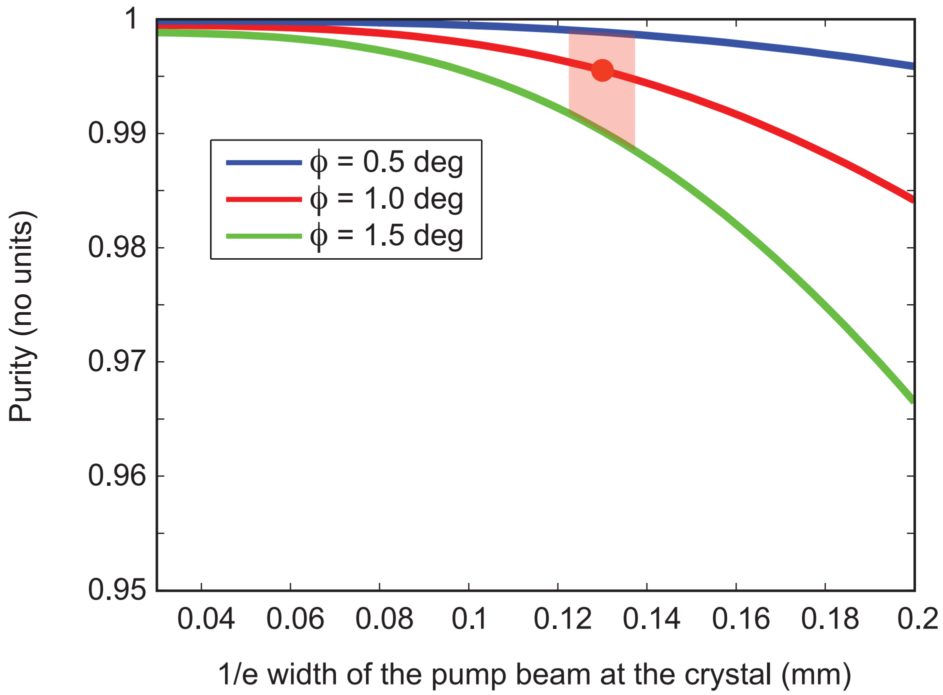

Supplementary Figure 1: **Purity of the spatial part of the SPDC field.** We measure the width of the pump beam as 0.13 ± 0.01 mm at the plane of the crystal and the collection angles as $\phi = 1.0 \pm 0.5$ degree for both collection modes. The red-colored region shows where the purity most likely lies, that is, between 0.99 and 1. To perform this calculation, which is in line with the formalism of reference [1], we used the parameters of our experiment: a pump spectrum with a 1/e bandwidth of 0.25 nm, spectral filters with 1/e bandwidths of 4 nm, collection modes with 1/e widths of 200 μm. For simplicity, the pump spectrum, the filter spectra and the collection special modes are all assumed to be Gaussian-distributed.

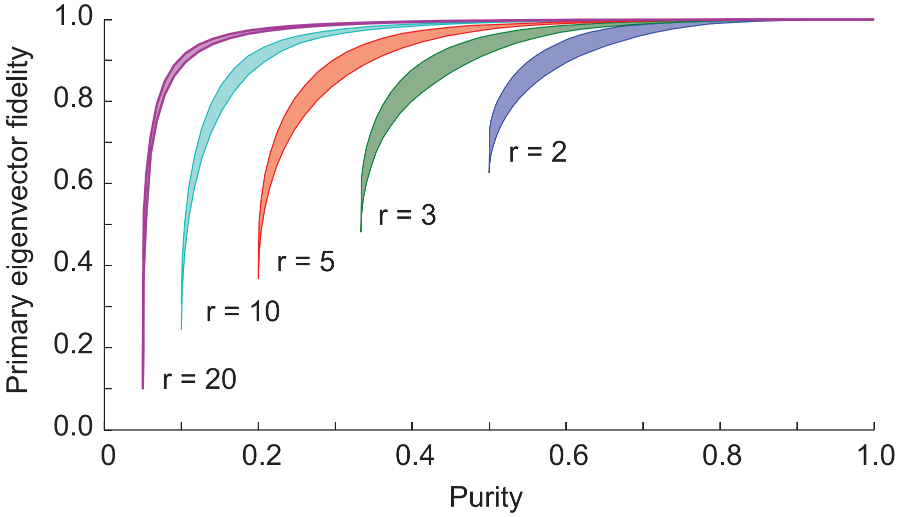

Supplementary Figure 2: **Numerical study of primary eigenvector recovery with the direct measurement approach in the case of mixed states.** We apply our direct measurement strategy to random density matrices of varying purity. For a given purity, we repeat the simulation 100 times and calculate the average fidelity between the primary eigenvector of the density matrix and the directly recovered state. For a given set of parameters, the fidelity most likely lies in the shaded area delimited by the hard colour lines, which represent one standard deviation from the mean. We perform this study for various ranks r of the density matrix. The simulations are performed for ranks of $r = \{2\ (\text{blue}), 3\ (\text{green}), 5\ (\text{red}), 10\ (\text{cyan}), 20\ (\text{purple})\}$. This result is nearly independent of the system dimensionality; the value of d is 40 for this simulation, but negligible variations occur for other valid dimensions ($d \geq r$).

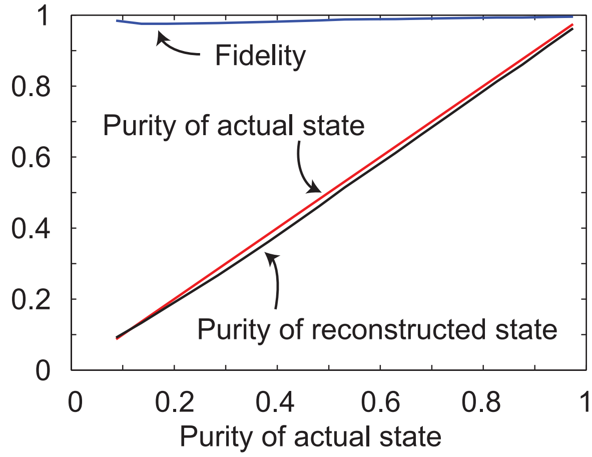

Supplementary Figure 3: **Simulation results of the density matrix recovery in the full tomography procedure.** We perform 5000 simulations using the projected gradient descent algorithm. The fidelity between the actual and recovered state (blue) is (0.986 ± 0.007) on average, where the uncertainty corresponds the standard deviation over all simulation runs. We generate (5×5)-dimensional density matrices with uniformly distributed purities; see red and black curves. We add Poissonian noise on the simulated data; the average error on a count rate is 2%. The parameters of the simulation are the following: $N = 1000$, $\gamma = 0.3$ and a number of iterations equal to 10000.

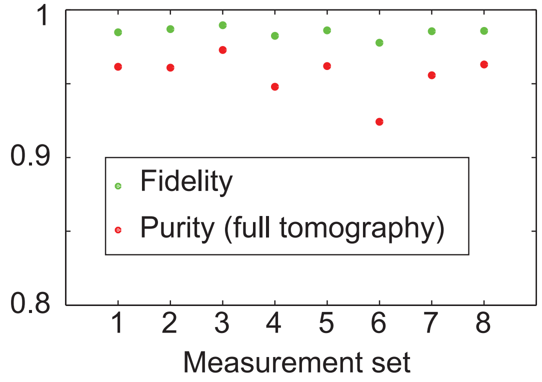

Supplementary Figure 4: **Comparison of recovered states with the direct approach and full tomography.** We perform the direct approach once and repeat the full tomography procedure eight times with the same subset dimensionality of (5×5) in the OAM space. The purity of the recovered density matrices is shown in red. We calculate the fidelity between the states obtained with the two methods (green) and obtain an average of (0.986 ± 0.007).

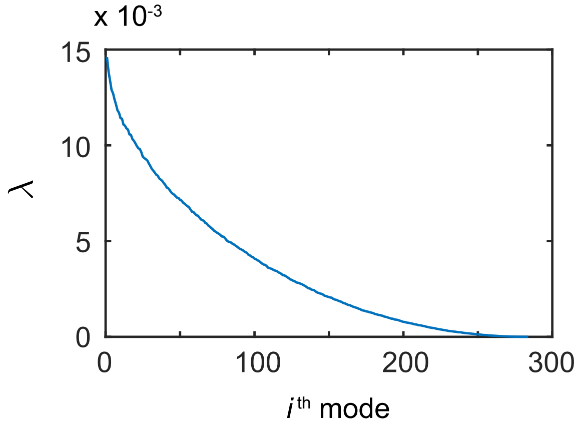

Supplementary Figure 5: **Probability of occurrence of the Schmidt modes calculated from the entire directly measured SPDC state vector.** We find a Schmidt number of 142 with the standard formula $1/\sum_i \lambda_i^2$ [10].

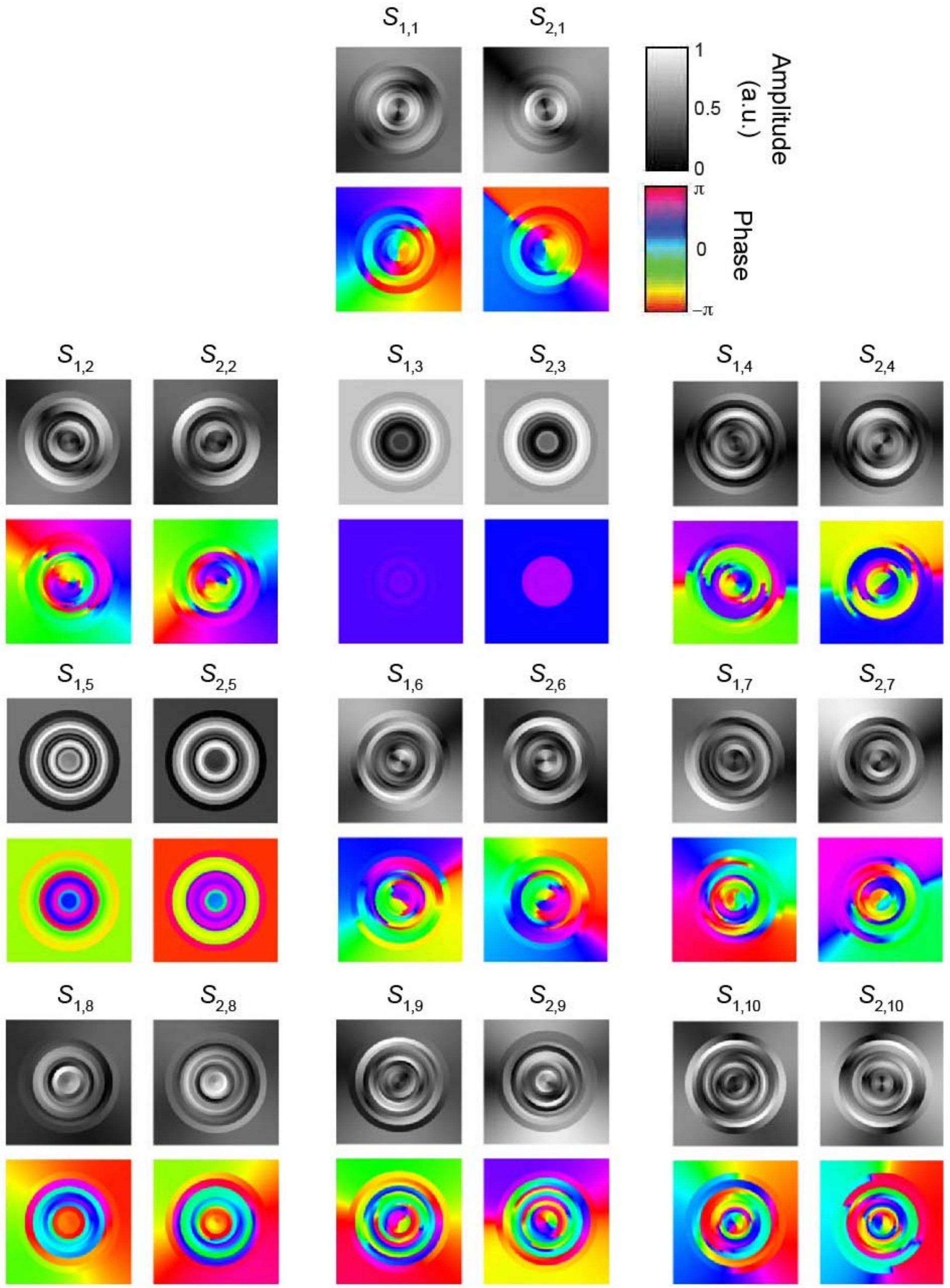

Supplementary Figure 6: **Amplitude and phase profiles of the first 10 Schmidt modes.** Before calculating the Schmidt modes, we process the data to reduce the contribution of the noise arising from the statistical nature of the measurements. When the amplitude of a given coefficient falls under a threshold of 0.04, we set the corresponding coefficient to zero. In principle, these theoretically calculated Schmidt modes form a basis in which the photons generated and detected in our experimental setup are highly correlated. The specific form of the Schmidt modes depends on the state generated via SPDC, the alignment of the setup and the geometry of the detection apparatus.

Supplementary Note 1. Purity of the spatial part of the SPDC state
In our experiment, we measure the spatial part of the two-photon field produced through SPDC, which needs to be pure for our method to remain exact. However, the SPDC process also gives rise to correlations between the frequencies Ω_1 and Ω_2 of the photons. The purity of the spatial part can only be guaranteed if the full state $|\Phi'\rangle$ can be written in the separable form

$$\begin{aligned}|\Phi'\rangle &= \int \mathbf{dk_1} d\Omega_1 \mathbf{dk_2} d\Omega_2 \Phi'(\mathbf{k_1}, \Omega_1, \mathbf{k_2}, \Omega_2)|\mathbf{k_1}, \Omega_1, \mathbf{k_2}, \Omega_2\rangle \\ &= \int \mathbf{dk_1} d\Omega_1 \mathbf{dk_2} d\Omega_2 H(\mathbf{k_1}, \mathbf{k_2}) G(\Omega_1, \Omega_2)|\mathbf{k_1}, \Omega_1, \mathbf{k_2}, \Omega_2\rangle,\end{aligned} \tag{1}$$

where $\mathbf{k_i}$ is the transverse wave vector of photon i.

One way to achieve separability is to project the frequency of each photon into very narrow bandwidth states, but this comes at the cost of lower count rates. A more efficient way to achieve separability is to work in the collinear regime [1]. The collinear regime is exactly attained when using a 50/50 beam-splitter to separate the photons and when the collection modes are centered in the middle of the SPDC field, but the beam-splitter leads to a 50% loss in efficiency. Instead, we separate the photons with a prism and make sure that the collection modes are as close to each other as possible. We calculate the purity of the SPDC state vector using the formalism of reference [1,2]. An important parameter is the average angle ϕ at which each photon comes out of the crystal with respect to the optical axis of the pump. We illustrate how the purity changes as a function of the deviation angle ϕ from the collinear regime in Supplementary Figure 1. From our experimental parameters, we estimate that the purity of the generated state in our experiment is greater than 0.99. Further to this, a method for directly measuring the purity of bipartite systems exists [5], but relies on non-local measurements, which we do not implement in our experiment.

Non-unit purity. When the state at hand is not pure, the outcome of the direct measurement procedure can yield valuable information for quite a large range of parameters. To demonstrate this, we perform Monte Carlo simulations on a variety of density matrices. We first choose the best reference vector $|j\rangle$ out of the computational basis $\{|1\rangle, |2\rangle, ..., |d-1\rangle\}$. This consists of making the measurements in this basis and using the state that yields the highest number of counts as the reference vector. We then simulate the direct measurement procedure without shot noise by taking the jth column of the density matrix and normalising it. We compute the primary eigenvector of the density matrix and calculate the fidelity with the normalised j^{th} column. We parametrise the density matrix ρ in the following way:

$$\rho = \lambda_0 |q_0\rangle\langle q_0| + \sum_{k=1}^{r-1} \frac{1-\lambda_0}{r-1} |q_k\rangle\langle q_k| \tag{2}$$

where $r > 1$ is the rank, λ_0 is the primary eigenvalue and $|q_0\rangle$ is the primary eigenvector. The purity of the density matrix is given by $\mathrm{Tr}[\rho^2] = \lambda_0^2 + (1-\lambda_0)^2/(r-1)$.

We perform the simulations for (5×5)-dimensional states for various purities and ranks; see Supplementary Figure 2. The lowest fidelity occurs when the density matrix is a mixture of two pure states ($r = 2$). In contrast, the results are more reliable when the eigenvalues are distributed amongst a high number of eigenvectors. The quality of the result depends mostly on the spectral gap of the density matrix, i.e. the difference between the primary eigenvalue and the second highest

eigenvalue. When this gap is high enough, the primary eigenvector is correctly recovered. Notably, when the purity is higher than 0.81, the fidelity is almost guaranteed to be higher than 0.99 in the absence of noise. This is true of any density matrix, independently of the chosen model. Given our parametrisation of the density matrix, the aforementioned spectral gap increases with rank, which explains why the fidelity also increases with rank.

Supplementary Note 2. Assumption-free tomography

In order to certify the validity of the direct measurement approach, we compare results obtained with the latter to density matrices acquired with full tomography. The full tomography procedure that we implement consists of minimising the objective function

$$f(\rho) = \left\| \frac{\mathcal{A}(\rho) - c}{\sqrt{\mathcal{A}(\rho)}} \right\|_2^2 \tag{3}$$

with the constraint that the density matrix ρ must be positive semi-definite. Here, $\mathcal{A}(\rho)$ is the vector of probabilities obtained with the guess density matrix ρ and c is the vector of experimentally obtained counts. To achieve this goal, we use a variation of the projected gradient algorithm of Candes *et al.* [3]. The term inside the 2-norm is derived through maximum likelihood analysis [4], and the division is performed element-wise. In the maximum likelihood analysis, the noise is assumed to be Gaussian with standard deviation equal to the square-root of the counts. Such Gaussian noise approximates Poissonian noise well in the presence of sufficiently high counts. We express the density matrix as a vector x of length d^2, and the distribution of the density matrix eigenvalues is taken to be a decreasing exponential. Similarly, we express the N measurement operators as vectors that we stack in a $(N \times d^2)$-dimensional matrix A. To reflect the experiment, each projector lies in a two-dimensional subspace of the total Hilbert Space. We iteratively converge to the density matrix that minimises the objective function by using its gradient

$$\nabla f(x) = 2A^\dagger \left[\left(\frac{Ax - c}{Ax} \right) \left(2 - \frac{Ax - c}{Ax} \right) \right], \tag{4}$$

where vector-vector operations are performed element-wise and matrix-vector operations are performed normally. The algorithm is given by: $x_k \leftarrow \mathcal{P}(x_{k-1} - \gamma \nabla f(x_{k-1}))$, where γ is a learning rate between 0 and 1. At each step, the operation $\mathcal{P}(\cdot)$ projects the new guess onto the set of positive semi-definite matrices by replacing all negative eigenvalues by zero. To reduce the number of required iterations, we start with the solution to the unconstrained problem, i.e. where x does not represent a physically realisable density matrix,

$$x_0 = (A^\dagger A + I/10)^{-1} A^\dagger c. \tag{5}$$

The identity term in the previous equation regularises the matrix to be inverted, such that x_0 depends less on the noise.

Note that this algorithm does not assume purity. We do not make an exhaustive theoretical analysis of the algorithm here, but we provide evidence for its validity by performing Monte Carlo simulations on 5000 quantum states of random purity; see Supplementary Figure 3. For an average number of counts of 2000 yielding a noise level $(\sum_i \sqrt{c_i}/c_i)/N$ of 2%, we find that the fidelity $\mathrm{Tr}[\sqrt{\sqrt{\rho}\rho_{\mathrm{act}}\sqrt{\rho}}]$ between the recovered density matrix ρ and the actual density matrix ρ_{act} is 0.98 on average.

We apply the projected gradient algorithm to the experimental data and find that the average fidelity between the directly measured state and the full tomography results of (0.985 ± 0.004); see Supplementary Figure 4. The average purity is (0.96 ± 0.02). These results clearly show consistency between the direct measurement approach and the full tomography method. The average fidelity between the directly measured state and the primary eigenvector of the recovered density matrix is (0.992 ± 0.002).

Supplementary Note 3. The Schmidt decomposition

In our system, we use the OAM-Walsh basis to perform measurements on entangled photon pairs. From a communications point of view, an error would occur when photon 1 is detected in mode $|\ell_1, k_1\rangle$ and photon 2 is detected in mode $|\ell_2, k_2\rangle$, where $\ell_1 \neq -\ell_2$ or $k_1 \neq k_2$ [6]. The off-diagonal elements in the probability matrix (Figure 1c and 1e) then correspond to the probability of getting an error. The basis $\{|S_1, S_2\rangle\}$ found via the Schmidt decomposition yields perfect correlations [7], thus theoretically removing any error. In the Schmidt basis, the measured SPDC state is given by

$$|\Phi_S\rangle = \sum_i \sqrt{\lambda_i}|S_{1,i}, S_{2,i}\rangle, \tag{6}$$

where λ_i are the probabilities associated with the Schmidt modes $|S_{1,i}, S_{2,i}\rangle$. We numerically find the Schmidt modes by performing a singular value decomposition on the directly measured SPDC state vector. We show the probability of occurrence λ_i of the Schmidt modes in Supplementary Figure 5 and illustrate, in Supplementary Figure 6, the first 10 Schmidt modes of the (33×33) subset from Figure 1b.

Supplementary Note 4. Positive operator value measure

In the case that POVMs can be applied to high-dimensional states, there is a strategy that combines the efficient measurement procedures afforded by POVMs and the direct approach to quantum state measurement. Any informationally complete set of L projectors $\{P_1, P_2, ..., P_L\}$ can be turned into a POVM with a simple recipe outlined in ref [8]: if one consider the matrix $G = \sum_k P_k$, then the set of POVM elements equal to $G^{-1/2}P_kG^{-1/2}$ forms a POVM. Using the informationally complete set of projectors from the main text for the above transformation, we can reconstruct every probability amplitudes from the measurement outcomes of POVM elements. Therefore there exists a POVM with which we can apply the entire direct measurement procedure.

Supplementary Note 5. Differential evolution algorithm

In order to find an efficient decomposition in terms of projective measurements, we run a differential evolution algorithm [9]. We set each vector element of the projectors and each complex weight as free parameters. We find the analytical solution by adjusting the parameters to the nearest rational numbers. The algorithm does not converge when we set the number of projectors to lower than five. This leads us to believe that five is the minimum number of local projectors for the exact construction of the two-body column-operator $\widehat{C}_{\ell_1,k_1}^{\ell_2,k_2} = |0,0\rangle\langle\ell_1, k_1| \otimes |0,0\rangle\langle\ell_2, k_2|$, where ℓ_1, k_1, ℓ_2, and k_2 are not equal to zero.

Supplementary References

[1] Osorio, C. I., Valencia, A. & Torres, J. P. Spatiotemporal correlations in entangled photons generated by spontaneous parametric down conversion. *New J. Phys.* **10**, 113012 (2008).

[2] Osorio, C. I. *Spatial Characterization Of Two-Photon States.* Ph.D. thesis, Univ. Politècnica de Catalunya (2014).

[3] Cai, J.-F. , Candes, E. J. & Shen, Z. A singular value thresholding algorithm for matrix completion. *SIAM J. Optimiz.* **20**, 1956–1982 (2010).

[4] James, D. F.V., Kwiat, P. G., Munro, W. J. & White, A. G. Measurement of qubits. *Phys. Rev. A* **64**, 052312 (2001).

[5] Bartkiewicz, K., Lemr, K. & Miranowicz, A. Direct method for measuring of purity, superfidelity, and subfidelity of photonic two-qubit mixed states. *Phys. Rev. A* **88**, 052104 (2013).

[6] Leach, J., Bolduc, E., Gauthier, D. J. & Boyd, R. W. Secure information capacity of photons entangled in many dimensions. *Phys. Rev. A* **85**, 060304 (2012).

[7] Ekert, A. & Knight, P. L. Entangled quantum systems and the Schmidt decomposition. *Am. J. Phys.* **63**, 415-423 (1995).

[8] Flammia, S. T., Silberfarb, A. & Caves, C. M. Minimal informationally complete measurements for pure states. *Found. Phys.* **35**, 1985–2006 (2005).

[9] Storn, R. & Price, K. Differential evolution – a simple and efficient heuristic for global optimization over continuous spaces. *J. Global Optim.* **11**, 341–359 (1997).

[10] Law, C. K., Eberly, J. H, Analysis and interpretation of high transverse entanglement in optical parametric down conversion. *Phys. Rev. Lett.* **92**, 127903 (2004).
